# Influence of the Surface Modification of Calcium Carbonate on Polyamide 12 Composites

**DOI:** 10.3390/polym12061295

**Published:** 2020-06-05

**Authors:** Fabio Ippolito, Gunter Hübner, Tim Claypole, Patrick Gane

**Affiliations:** 1 Research and Development Department, Omya International AG, 4665 Oftringen, Switzerland; 2 Department of Printing and Media, Hochschule der Medien, 70569 Stuttgart, Germany; huebner@hdm-stuttgart.de; 3 College of Engineering, Swansea University, Swansea SA2 8PP, UK; t.c.claypole@swansea.ac.uk; 4 Department of Bioproducts and Biosystems, Aalto University, 02150 Espoo, Finland; patrick.gane@aalto.fi

**Keywords:** composite, limestone, calcium carbonate, polyamide 12, mechanical properties

## Abstract

In previous investigations, it was found that the thermal properties of a polyamide 12 compound can be manipulated, using a designed filler, to improve the melting as well as crystallization behavior, determined for selective laser sintering. A common downside of the introduction of a non-flexing mineral filler is the reduction of the mechanical properties, such as ductility. This paper investigates the influence of content and surface modification of limestone on the mechanical properties. The aim is to understand the effect of an optimized coupling agent on the properties of a compound, containing polyamide 12 filled with 10 wt % of surface modified calcium carbonate. A range of four mineral filler modifications was chosen to investigate their coupling effect, namely 6-amino hexanoic acid, *ε*-caprolactam, l-arginine or glutamic acid. The in advance surface modified fillers were then each used in combination with the polyamide 12 in a twin-screw extrusion process. With an optimized surface modifying agent, the tensile strength as well as elongation at break can be improved in comparison with uncoated filler implementation, such that up to 60% of the loss of ductility and toughness of a final part when using an untreated filler could be regained using an optimized surface modifier at a correct amount. With the tested filler grade and the specific tested filler amount, the optimized amount of 6-amino hexanoic acid was approx. 2.5 mmol of treatment agent per 100 m^2^ of CaCO_3_. These found improvements in a twin-screw extruded polyamide 12 compound show the possible usage of modified calcium carbonate as a functional filler in additive manufacturing and can potentially be transferred in a subsequent investigation in the selective laser sintering process.

## 1. Introduction

In the additive manufacturing (AM) process, a three-dimensional object is printed layer by layer, in which each layer is bonded/fused together by several different techniques (sintering, melting, chemical reaction, curing, etc.) [1]. Such AM technologies are able to build complex geometries, which are considered difficult, or even impossible, to realize using subtractive techniques [2].

AM technologies have been introduced in various application areas, such as aerospace, automotive, artistic design and biomedicine [3,4,5,6]. Materials commonly used in AM are often plastic or metal based [7,8].

Selective laser sintering (SLS) is an additive manufacturing process, in which a computer-aided design (CAD) drawing is printed out of a powder bed by fusing the layers together by a laser beam of a suitable material-absorbing wavelength. Various common engineering thermoplastics or metals are used [9]. The most frequently used thermoplastics are polyamides/nylons, typically polyamide 12.

During the past years, many investigations have been carried out to define the limitations and most crucial factors, which can prevent successful processing through selective laser sintering [10,11,12,13,14,15,16]. The most important influence can be attributed to the variable thermal properties as well as particle size of the polymer/composite powder [17]. Y. Guo et al. introduced the concept of reducing the manufacturing and material costs, as well as improving energy efficiency, by blending the polymer matrix with an environmentally friendly filler material, such as calcium carbonate [18]. This also addresses the environmental issue and high energy consumption, derived with selective laser sintering [19,20,21].

Introducing filler material into the polymer matrix, with different thermal capacity as well as thermal conductivity, can be beneficial. Higher thermal conductivity (than polyamide 12 alone) could reduce the laser power needed to sinter the polymer, whilst the greater thermal capacity offered by the carbonate could result in the ability to use a higher total energy density without resulting in curling during the crystallization step [22]. A further consideration could be to use a filler concept that enhances laser radiative absorption.

As shown in previous investigations, limestone as a functional filler can be adjusted to improve the melting as well as crystallization behavior, manipulated for an improved selective laser sintering process [22]. Such an introduction of a rigid mineral filler results in a loss of mechanical properties, which is not desirable.

Besides an optimal particle size and morphology of the composite, the chemical interaction between the composite fractions can be employed to advantage, or conversely can be manifest as a disadvantage, influencing both processability (homogeneous distribution and sinterability) as well as final properties. The chemical interaction between an additive and the surrounding polymer matrix can be improved by an adjusted chemical coating of the additive. This results in an increased processability as well as final properties of a produced element.

The adhesion between filler and a polymeric material is usually weak due to the poor compatibility of the polymer with the filler particle surface. In the case of calcium carbonate filler, as an example of mineral filler, to improve the compatibility between the nitrogen groups within the polyamide 12 chain and the calcium carbonate surface, a precise adhesion promoter can be implemented. As suggested by H. Goodman, a surface coating presenting amino acids, for example 6-aminocaproic acid, results in an improved bonding to the polar groups within the polymer structure, hence improving the mechanical properties [23]. As suggested by the literature, the carboxylic acid group of a modifier agent can react with a hydroxyl group on the calcite surface in a condensation reaction, hence, in this case, resulting in free amino groups on the bound molecule at the surface of the mineral filler material [24]. Through such a surface modification, hydrogen bonding to an external material can take place, which improves the adhesion between the filler and the polymer matrix.

With this antecedent study to improve a mineral filler by surface modification and investigating the potential improvement on the final properties of a twin-screw extruded compound, the results reported here on a best-suited surface modifier as well as modification amount can be transferred onwards to a successional investigation in an actual additive manufacturing process.

## 2. Materials and Methods

The first step to determine the suitability of a new type of compound for selective laser sintering (SLS) is to compare the thermal and mechanical properties of compression molded compound with those of the virgin polymer.

The thermal and flow properties of a polyamide 12 matrix can potentially be manipulated to improve a laser thermal sintering process insofar as the layer melting process can be optimized as well as the crystallization process accelerated [22].

In this study we set out to establish the beneficial role of an optimized surface modification of our mineral filler, compounded with polyamide 12 in respect to the resulting mechanical properties.

To be able to compare the produced compounds with the results from previous investigations, the compression molding process, as well as the composite analysis were kept identical as described by F. Ippolito et al. [22]. For completeness, these methods are again outlined in the following sections.

### 2.1. Materials

The mineral filler used for this study was commonly applied calcium carbonate (GCC), in this case ‘Omyafilm 753–OM untreated’, a marble-derived product provided by Omya International AG (Baslerstrasse 42, 4665 Oftringen, Switzerland).

SLS-approved polyamide 12 (PA2200) powder was obtained from EOS e-Manufacturing Solutions (Electro Optical Systems, Robert-Stirling-Ring 1, 82152 Krailling, Germany).

**Figure 1:**
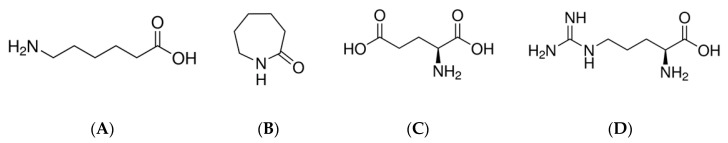
Molecular structure of (**A**) 6-amino hexanoic acid; (**B**) *ε*-caprolactam; (**C**) glutamic acid and (**D**) l-arginine.

General material specifications are listed in Table 1. For the surface modification agent, a selection of various amino-containing acids was chosen to improve the compatibility of the mineral filler material with the polar group in the polymer structure. The defined amino acids were chosen to be able to determine a difference in the polymer interactional compatibility of end- against side-chained amino groups. The molecular structure of each amino acid surface modifier is shown in Figure 1.

As benchmark, stearic acid, a typical surface modifier for calcium carbonate in polyolefin applications, was used [25].

General material specifications for the surface modifiers are listed in Table 2.

### 2.2. Functional Filler Production

The modifications of the raw filler material were made using a dry pigment surface modification process. To develop a homogeneous treatment, a batch coater (MP-LB mixer from Somakon Verfahrenstechnik UG, 44536 Lünen, Germany) was used, in which the mineral filler was pre-heated up to 120 °C and a stirring speed of 500 min^−1^ (rpm). The coating agent was distributed under conditions of uniform heating and homogeneous mixing for 10 min maintaining the temperature at 120 °C. Table 3 shows an overview of all carried out filler surface modifications and the modifier used.

### 2.3. Composite Manufacturing

#### 2.3.1. Preparation of Formulations

The solid polyamide 12 powder was homogeneously premixed with the specific weight-defined amount of surface-coated functional filler to achieve 10 wt % filler loading. This powder processing resulted in a homogeneously mixed powder blend, for use directly in compression molding.

Since the interaction occurs at the interface between the filler and the polymer, surface area was used to describe the functional loading effect of the filler per given mass of polymer. In this case, the introduced surface area of filler per given mass of polymer at the designed loading of 10 wt % was 41.1 m^2^ filler/100 g PA12.

#### 2.3.2. Compounding

Compounds were extruded via a twin-screw extruder system (Extruder ZE 12 from Three-Tec GmbH, 5703 Seon, Switzerland). The extruder barrel-length was 25 cm and the twin-screws had a diameter of 12 mm with a flank pitch of 12 mm. The barrel temperature profile was split in three parts along its length, with an inlet temperature of 160 °C, a compounding zone at 200 °C and an outlet temperature of 170 °C. The twin-screw rotation speed was kept constant for all trials at 90 min^−1^ (rpm), resulting in a compound residence time of approximately 30 s. The compound was formed through a filament nozzle with a diameter of 2 mm and granulated into cylindrical pellets with a length ≈ 1 mm.

**Figure 2:**
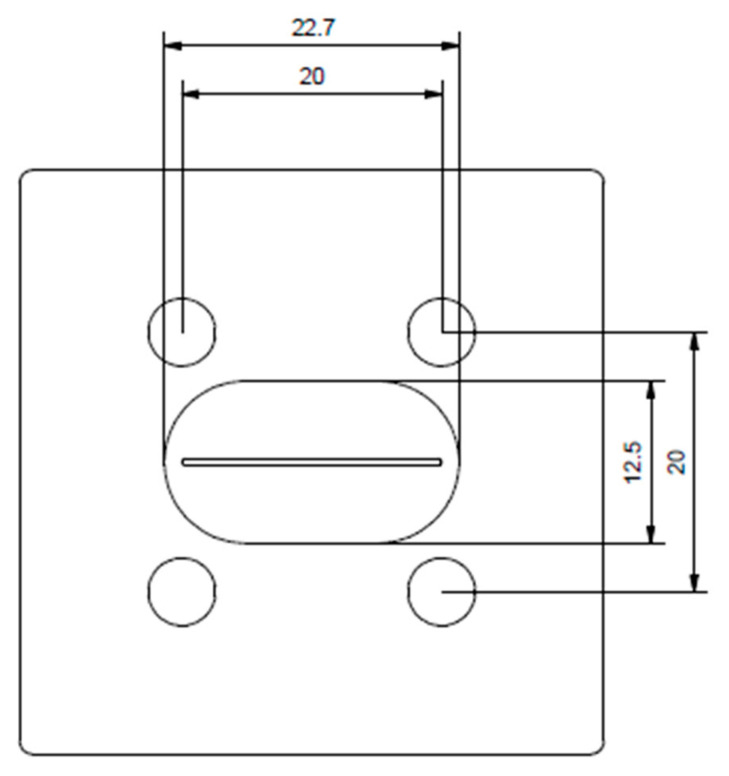
Plate nozzle used for plate extrusion (dimension in mm).

**Figure 3:**
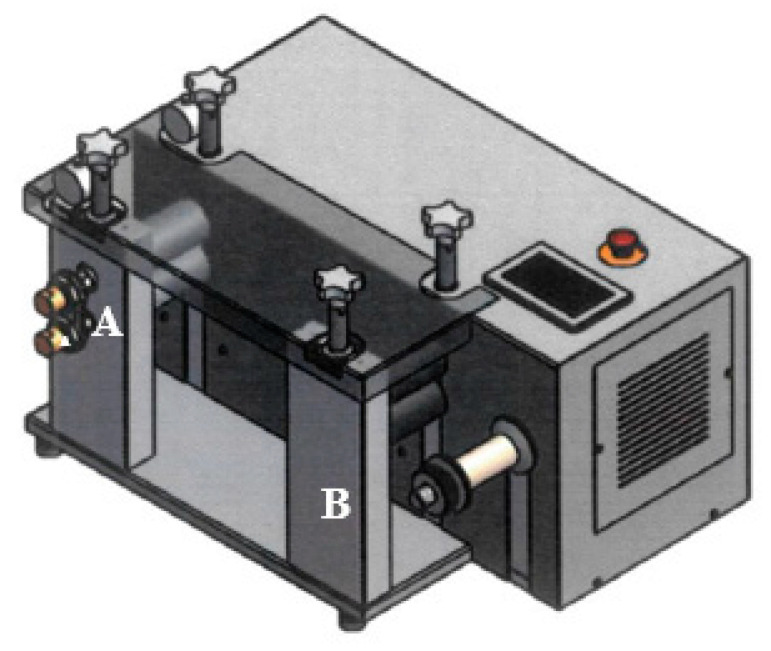
Graphical layout of used calender system with the (**A**) calender press and the (**B**) extract roller.

In addition to the pellets, approx. 1-mm-thick sample plates with a width of 3 cm were produced. The compound was formed in this case through a plate nozzle (Figure 2) with a thickness of 2 mm and a width of 2 cm, passed through a calender press and pulled by an extract roller. The calender was set to a thickness of 1 mm and a roller speed adjusted to the extruder. Figure 3 shows a schematic of the used calender system.

**Figure 4:**
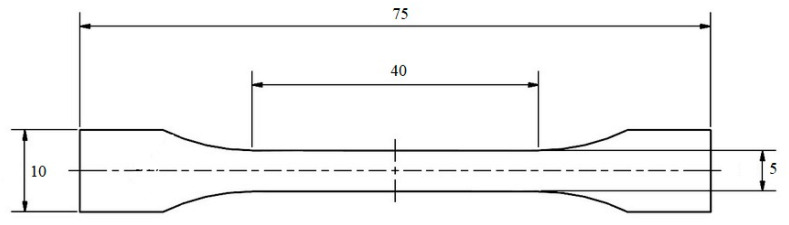
Dog bones used for tensile tests (dimension in mm).

The resulting plates were punched to normalized tensile test ‘dog bones’ for further analysis (Figure 4).

The compounded samples were stored at a constant relative humidity of 50% at a temperature of 22 °C for at least 24 h before analysis.

### 2.4. Composite Analysis

#### 2.4.1. Scanning Electron Microscopy

The uniformity of compounding, with emphasis on the homogeneity of filler distribution within the polymer/filler matrix, was observed using scanning electron microscopy (SEM) (Zeiss Sigma VP, Carl-Zeiss-Strasse 22, 73447 Oberkochen, Germany).

The pellets were pressed above the melting point at a temperature of 210 °C onto a steel metal plate with a thickness of ≈ 2 mm. The pressed compound samples were embedded in epoxy resin and SEM specimens were prepared by cutting to form a planar internal surface with a diamond knife of 20 μm and afterwards with 15 μm thickness and polishing with corundum (0.05 μm). The specimens were studied with a field emission SEM in variable pressure mode (50 Pa) at 20 kV and a 60-μm cover.

#### 2.4.2. Thermogravimetric Analysis

To confirm the homogeneity of filler distribution, the extruded compounds were analyzed by thermogravimetric analysis (TGA) (Im Hackacker 15, 8902 Urdorf, Switzerland).

The TGA technique is used to identify changes in physical and chemical properties of materials as a function of increasing temperature. It is regularly used to measure the thermal degradation of filled organic materials. Partially, it is possible to determine reliable approximate values concerning the humidity, the content of low-molecular auxiliary agents and the organic content. Depending on the measuring method, the thermal degradation can be determined up to 1000 °C as a summation (cumulative loss) parameter.

TGA curves were recorded on a Mettler-Toledo TGA/DSC 1-LF apparatus under a continuous nitrogen flow of 50 cm^3^ min^−1^. The samples (≈ 150 mg) were heated to 100 °C, kept at constant temperature for 2 min and heated up to 700 °C at a rate of 10 °C min^−1^. The gravimetric weight loss of the compound during temperature increase from 100 up to 600 °C was measured.

#### 2.4.3. Mechanical Properties

The introduction of ground calcium carbonate as mineral filler material into a polymeric system is generally known to result in a significant drop in material ductility, i.e., the compound becomes brittle [26]. By improving the adhesion force between the filler and polymer matrix, this loss in ductile behavior can be reduced, hence improving the overall functionality of calcium carbonate filler in polyamide.

#### 2.4.4. Tensile Test

The mechanical properties of each compounded sample were evaluated with a Zwick/Roell ProLine table-top testing machine (ZwickRoell GmbH & C. KG, August-Nagel-Strasse 11, 89079 Ulm, Germany) according to the European Norm ISO 527-1 guidelines. Ten tensile test dog bones of each compound were measured adopting a tensile extension rate of 1 mm min^−1^ providing a determination between 0.05% and 0.25% extension. Afterwards, the yield stress was measured with a rate of 50 mm min^−1^, with a force threshold of 60% of the maximum force and a sensitivity of 0.5%.

## 3. Results and Discussion

### 3.1. Homogeneity of Filler Distribution

#### 3.1.1. Morphology

**Figure 5:**
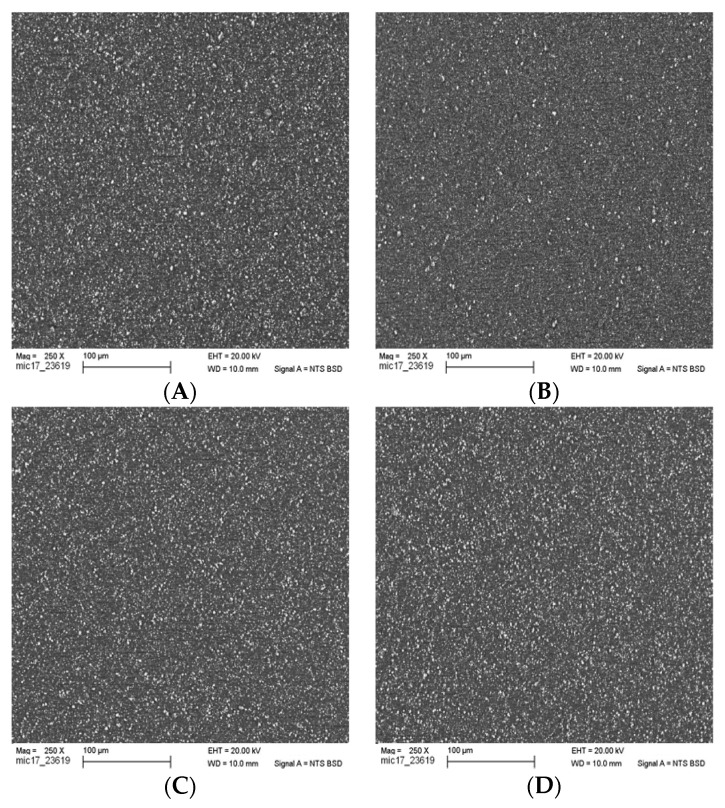
SEM images of morphology of PA-CaCO_3_ composites: (**A**) 1% amino hexanoic acid treated; (**B**) *ε*-caprolactam treated; (**C**) l-arginine treated; (**D**) glutamic acid treated.

The SEM pictures in Figure 5 show the elemental contrast within the sample, where the filler materials reflect more electrons and appear brighter (white spots on the image) than the polymer matrix, which constitutes the black (electron-absorbing) background. The degree of the compounding homogeneity at the loading level of 10 wt % filler can be seen across the different filler surface modifications with the different amino acids (A) amino hexanoic acid, (B) *ε*-caprolactam, (C) l-arginine and (D) glutamic acid.

The filler material is indeed homogeneously distributed throughout the polymer matrix as exemplified in all four cases. The change of the surface modification agent showed no significant influence on filler particle–particle interaction, i.e., not worsening the agglomeration behavior.

#### 3.1.2. Filler Amount Implementation

**Figure 6:**
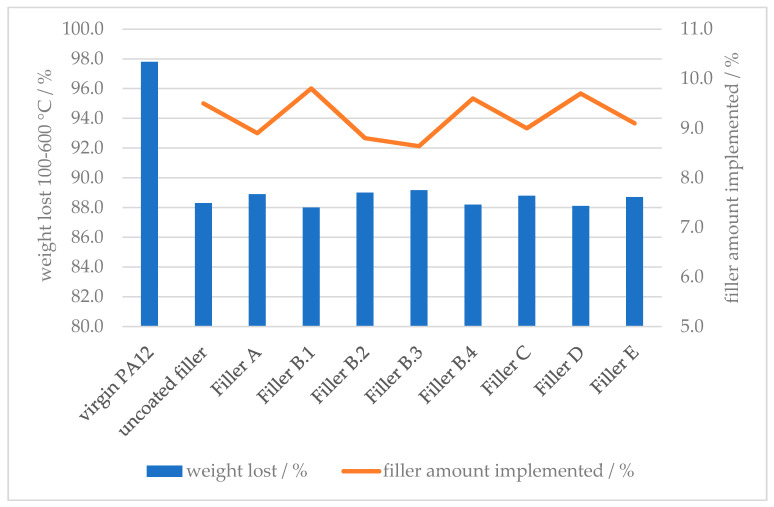
Thermogravimetric Analysis—weight loss and filler amount implementation.

The actual implemented amount of mineral filler can be detected by the difference of the loss of organic compound, in comparison with the virgin polymer (Figure 6). The actual compounded amount of mineral filler throughout all samples were in the range of 9.2 ± 0.7 wt %.

In combination with the SEM results, the filler implementation for all tested functional fillers can be considered as homogeneously distributed and consistent. Differences in the mechanical properties can therefore be traced back uniquely to the influence of the surface modification of the filler.

### 3.2. Mechanical Properties

#### 3.2.1. Influence on Stiffness/Tensile Modulus

As indicated by Y.W. Leong et al., the implementation of a rigid filler in a polymer matrix, results in an increase of the tensile modulus, due to increase in stiffness and the restriction of the mobility and deformability of the matrix [26].

**Figure 7:**
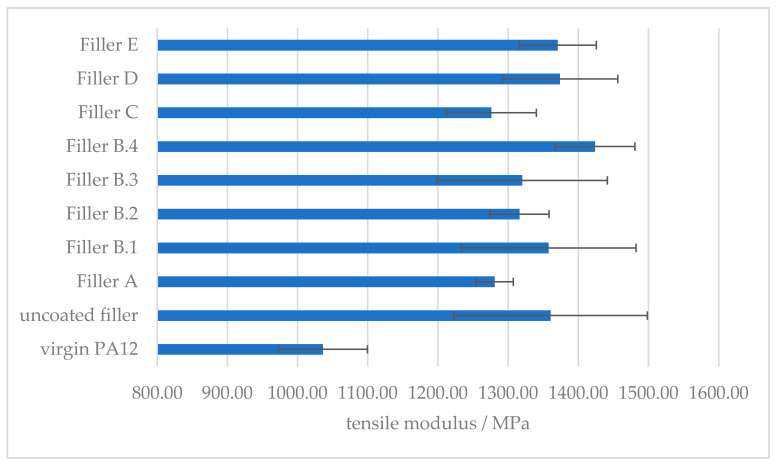
Effect of the amount of surface modifier on the tensile modulus of amino hexanoic acid treated CaCO_3_-filled PA12 composites.

The tensile modulus can be increased by approx. 30% with the introduction of the approx. 40 m^2^ filler/100 g PA12 (Figure 7). The tensile modulus is measured before any significant plastic deformation takes place, so is not influenced by the interaction between the filler and the polymer matrix [26]. Therefore, this increase in stiffness stays unaffected by the surface modification of the mineral filler with the different amino acids, as long as the filler amount is consistent.

**Figure 8:**
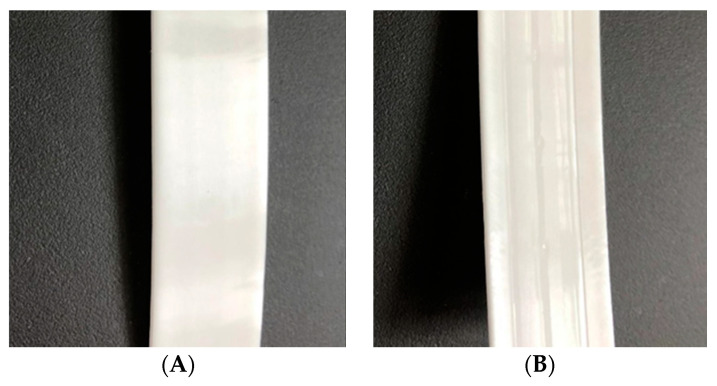
Extruded plate with (**A**) smooth front side and (**B**) back side with slight inhomogeneity.

The measured deviation between the 10 tested “dog bones” of each sample is quite high. This is a result of the experimental set up, used for these investigations. Due to the direct plate extrusion of the compound via a twin-screw extruder and a calender system, the outer layer of the plates shows a slight inhomogeneity. This effect can be seen in Figure 8 and is the same throughout all produced samples. Since the punched out “dog bones” are quite thin and small, this effect and the resulting high deviation on the modulus is significantly higher than it is on properties after plastic deformation takes place.

This increased deviation could be reduced by injection molding larger and thicker tensile test pieces, instead of a direct plate extrusion process.

#### 3.2.2. Influence on Ductility and Toughness

##### Effect of Surface Modifier Chemistry

**Figure 9:**
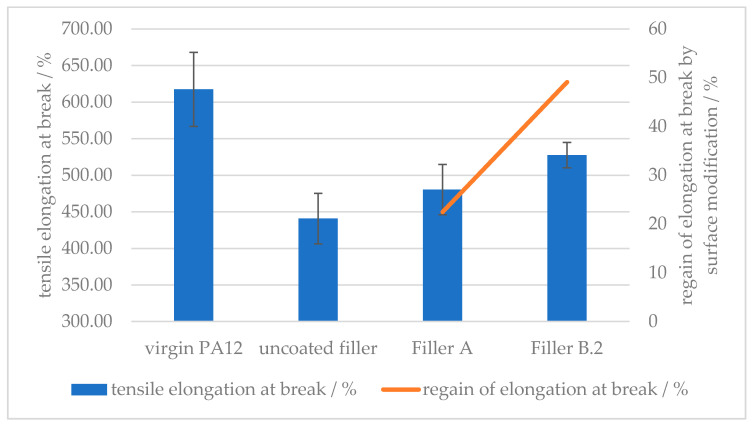
Effect of the surface modifier chemistry on the elongation at break of (**A**) stearic acid and (**B**) amino hexanoic acid treated CaCO_3_-filled PA12 composites.

The influence of the modifier chemistry was compared to prove that by the implementation of free amino groups, bound on the surface of the mineral filler material, hydrogen bonding can take place, hence improving the adhesion between the filler and the polymer matrix. Figure 9 compares the increase in the elongation at break of an unsuited surface modifier, namely stearic acid, with the effect of the same amount of amino hexanoic acid. By optimizing the surface modification, hence increasing the intermolecular forces between the filler material and the polymer, the elongation at break can be improved from 440% up to 530 %. This results in a regain of 50% of the lost ductility by implementing a solid mineral filler. The use of stearic acid as a surface modifier results in a regain of 20% of the lost properties, due to the plasticizing effect of stearic acid, embedded into the polymer matrix [27].

**Figure 10:**
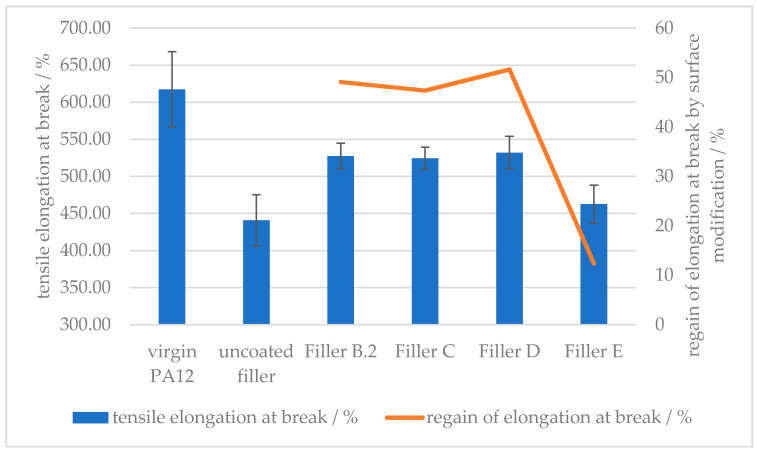
Effect of the surface modifier chemistry on the elongation at break of (**B**) amino hexanoic acid; (**C**) *ε*-caprolactam; (**D**) l-arginine; (**E**) glutamic acid treated CaCO_3_-filled PA12 composites.

**Figure 11:**
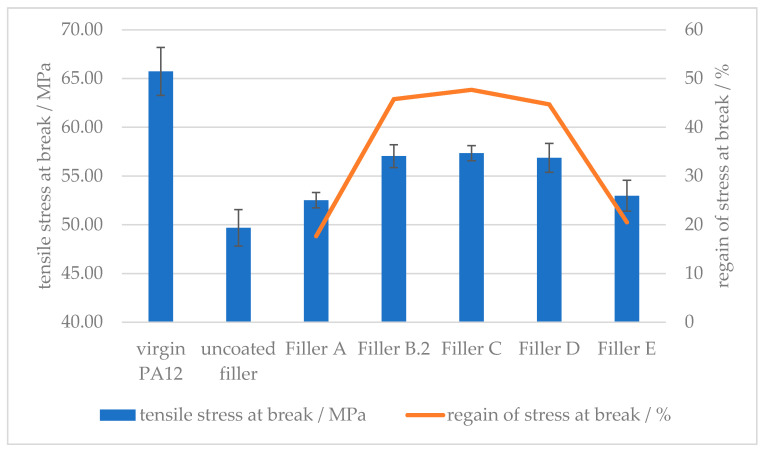
Effect of the surface modifier chemistry on the tensile stress at break of (**A**) stearic acid, (**B**) amino hexanoic acid; (**C**) *ε*-caprolactam; (**D**) l-arginine; (**E**) glutamic acid treated CaCO_3_-filled PA12 composites.

Viewing Figure 10 and Figure 11 allows a comparison to be made between the different amino acid types used in this study. The surface modification with the same amount of amino hexanoic acid and *ε*-caprolactam showed similar behavior with regards to elongation at break. The cyclic structure of caprolactam breaks open and the carboxylic acid group binds the same way on the calcium carbonate surface as amino hexanoic acid. Although l-arginine has more free nitrogen groups, which could increase the hydrogen bonding forces between filler and polymer matrix, the increased regain of elongation at break, in comparison with 6-Amino hexanoic acid, is negligible. This can be explained by comparing the influence of side-chained amino groups on the elongation at break. With a surface modification with glutamic acid, which only possesses a side-chained amino group, the regain of force at break drops below 20%, as for l-arginine, which consists of an end-chained as well as side-chained amino groups, the regain is at 50%. The same regain of force at break counts for a corresponding amount of 6-amino hexanoic acid, which only offers an end-chained amino group. Indicating the hydrogen bonding forces of side-chained amino groups are too weak to result in an increase of the ductility, if used as surface modifier for mineral fillers in a polyamide 12 matrix.

##### Effect of Surface Modifier Amount

Determination of the ductility and toughness as a function of the filler surface modifier amount indicates a clear dependency of the achievable regain of the reduced properties.

**Figure 12:**
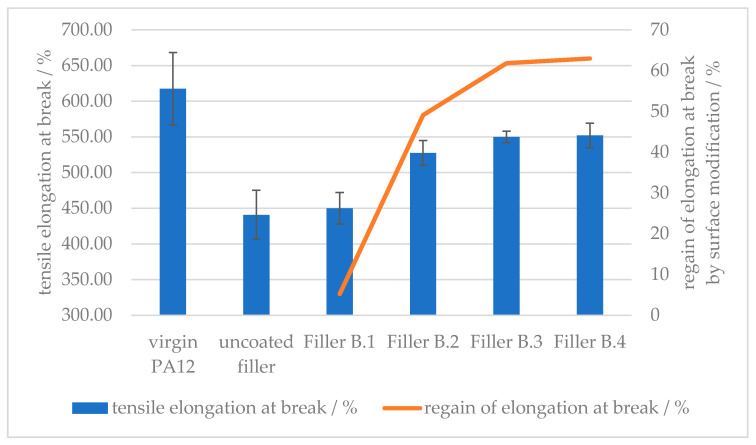
Effect of the amount of surface modifier on the tensile elongation at the break of amino hexanoic acid-treated, CaCO_3_-filled PA12 composites.

Figure 12 shows how the elongation at break is reduced by approx. 30% by the implementation of the given amount of calcium carbonate as mineral filler. With increased treatment amount of 6-amino hexanoic acid, hence increased polar bonding groups on the filler surface, the ductility can be regained up to approx. 60% of the filler-free value. The difference plateaus between 1.5 and 2.0 wt % of surface modifier on the mineral filler, suggesting that the optimum amount of 6-amino hexanoic acid as the surface modifier is between 1.0 and 1.5 wt %.

**Figure 13:**
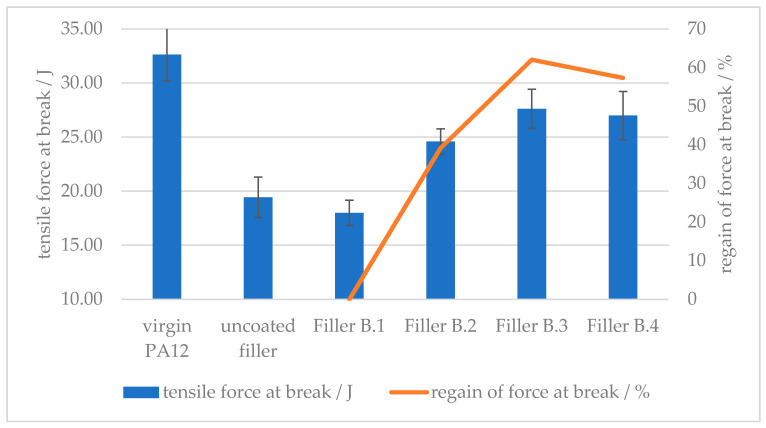
Effect of the amount of surface modifier on the tensile force at break of amino hexanoic acid treated CaCO_3_-filled PA12 composites.

A similar effect can be seen in Figure 13 in respect to the tensile force at break. With up to 1.5 wt % of Amino hexanoic acid as filler surface modifier, the loss in tensile toughness can be regained up to approx. 60% in comparison with an uncoated filler implementation.

## 4. Conclusions

Calcium carbonate–polyamide 12 composites have been produced via twin-screw compounding using calcium carbonate as filler material, surface modified with different modification agents, namely 6-amino hexanoic acid, *ε*-caprolactam, l-arginine or glutamic acid.

It was seen that the surface modifier chemistry as well as the amount of modifier agent have an influence on the mechanical properties with respect to the ductility, toughness and stiffness of the final part, which can be summarized as follows:

- The tensile modulus of a compound can be increased by approx. 30 wt % with the introduction of approx. 40 m^2^ filler per 100 g of PA12.
  - This effect on the stiffness is unaffected by the surface modification of the mineral filler, since the tensile modulus is measured before any plastic deformation takes place.
- The ductility as well as tensile strength shows a clear improvement, if the filler is surface modified with an appropriate amino acid instead of stearic acid
  - The greatest improvements were obtained with amino acids, which consist of free amino groups at the end of the carboxylic chain, instead of only as side chains.
  - To have an optimized filler/coated adhesion promoter ratio, between 1.0 and 1.5 wt % of surface modifier with respect to calcium carbonate is needed. With the tested filler grade, this results in an approx. optimum of 2.5 to 3.0 mmol of treatment agent per 100 m^2^ CaCO_3_.

Up to 60% of the loss of ductility of a polyamide 12 matrix, following from the introduction of a non-flexible mineral filler, can potentially be regained with appropriate surface modification of the filler.

These improvements in a twin-screw extruded polyamide 12 compound show the potential of modified calcium carbonate as a functional filler, and, when combined with earlier work showing the impact of particle size and filler quantity on thermal conductivity and crystallization within the polymer matrix, it now follows that such a combination could provide an optimized development pathway in additive manufacturing and can be transferred in a subsequent investigation in the selective laser sintering process.

## Figures and Tables

**Table 1:** Material specification.

	Omyafilm 753–OM	PA2200
producer/supplier	Omya International	EOS e-Manufacturing
volume-based median particle size	2 μm	60 μm
particle shape	irregular	spherical
approx. thermal conductivity at 298 K	1.3 Wm^−1^ K^−1^	0.2 Wm^−1^ K^−1^
approx. specific heat	0.8 kJkg^−1^ K^−1^	1.2 kJkg^−1^ K^−1^
specific surface area (BET)	3.7 m^2^ g^−1^	

**Table 2:** Material specification of surface modification agents.

	Stearic Acid	Amino Hexanoic Acid	*ε*-Caprolactam	l-Arginine	Glutamic Acid
producer/supplier	Wilfar	Sigma Aldrich	Sigma Aldrich	Sigma Aldrich	Sigma Aldrich
CAS Number	57-11-4	60-32-2	105-60-20	74-79-3	56-86-0
Linear Formula	C_18_H_36_O_2_	C_6_H_12_NO_2_	C_6_H_11_NO	C_6_H_14_N_4_O_2_	C_5_H_9_NO_4_
Molecular weight	284.5 g mol^−1^	131.17 g mol^−1^	113.2 g mol^−1^	174.2 g mol^−1^	147.1 g mol^−1^

**Table 3:** Resulting functional filler data after drying step.

Filler Definition	Surface Modifier	Additive Amount/% by Weight (wt %)
**A**	Stearic acid	1.0 ± 0.1
**B.1**	Amino hexanoic acid	0.5 ± 0.1
**B.2**	Amino hexanoic acid	1.0 ± 0.1
**B.3**	Amino hexanoic acid	1.5 ± 0.1
**B.4**	Amino hexanoic acid	2.0 ± 0.1
**C**	*ε*-Caprolactam	1.0 ± 0.1
**D**	l-Arginine	1.0 ± 0.1
**E**	Glutamic acid	1.0 ± 0.1

## Supplementary Materials

The following are available online at https://www.mdpi.com/2073-4360/12/6/1295/s1.
