# Supplementary material for: Influence of the Surface Modification of Calcium Carbonate on Polyamide 12 Composites
Source: Polymers (Basel). 2020 Jun 5;12(6):1295. doi: 10.3390/polym12061295 (PMC7362199; doi:10.3390/polym12061295)

# Zugprüfung ISO 527
# Probekörper 1BA (1:2)

16.05.19

FOK-Nummer : 172314/00A
Rezeptur-Nr. : unfilled
Kunde :
Prüfer : IPF
Material : EOS PA2200
Füllstoff :
Maschinendaten : Steuerung WN: 158988
Traverse WN: 158988
Kraft 20 kN WN: 158989
Makro-Wegaufnehmer WN: 162140

Vorkraft : 2 N
Geschwindigkeit Zugmodul : 1 mm/min
Prüfgeschwindigkeit : 100 mm/min
Beginn Zugmodulermittlung : 0.05 %
Ende Zugmodulermittlung : 0.25 %
Messlänge Standardweg : 25 mm

## Prüfergebnisse:

| Nr | Breite B mm | Dicke A mm | E-Modul N/mm² | Streckspannung N/mm² | Streckdehnung % | Zugfestigkeit N/mm² | Dehnung Zugf. % | Bruchspannung N/mm² | Bruchdehnung % | W bis Bruch J |
|---|---|---|---|---|---|---|---|---|---|---|
| ✝ 1 | 5 | 0.98 | 1180 | 39.3 | 5.8 | 54.7 | 414.9 | 54.2 | 415.3 | 18.68 |
| 2 | 5 | 0.98 | 1110 | 39.3 | 5.9 | 72.9 | 682.3 | 68.8 | 689.2 | 38.47 |
| 3 | 5 | 0.98 | 1130 | 39.0 | 6.0 | 65.9 | 604.4 | 64.8 | 615.8 | 32.58 |
| 4 | 5 | 0.98 | 910 | 38.5 | 6.1 | 73.0 | 660.0 | 72.2 | 661.5 | 37.00 |
| 5 | 5 | 0.98 | 1130 | 38.2 | 6.2 | 60.1 | 569.4 | 59.5 | 570.0 | 27.47 |
| 6 | 5 | 0.98 | 879 | 38.7 | 6.3 | 65.4 | 609.3 | 65.4 | 609.3 | 32.02 |
| 7 | 5 | 0.98 | 1060 | 39.1 | 6.1 | 63.6 | 558.3 | 63.6 | 558.8 | 28.23 |

## Statistik:

| Serie n = 6 | Breite B mm | Dicke A mm | E-Modul N/mm² | Streckspannung N/mm² | Streckdehnung % | Zugfestigkeit N/mm² | Dehnung Zugf. % | Bruchspannung N/mm² | Bruchdehnung % | W bis Bruch J |
|---|---|---|---|---|---|---|---|---|---|---|
| $\bar{x}$ | 5 | 0.98 | 1040 | 38.8 | 6.1 | 66.8 | 614.0 | 65.7 | 617.5 | 32.63 |
| s | 0.000 | 0.000 | 113 | 0.402 | 0.1 | 5.16 | 49.0 | 4.37 | 50.7 | 4.46 |
| ν | 0.00 | 0.00 | 10.94 | 1.04 | 2.41 | 7.73 | 7.97 | 6.65 | 8.21 | 13.68 |

## Seriengrafik:

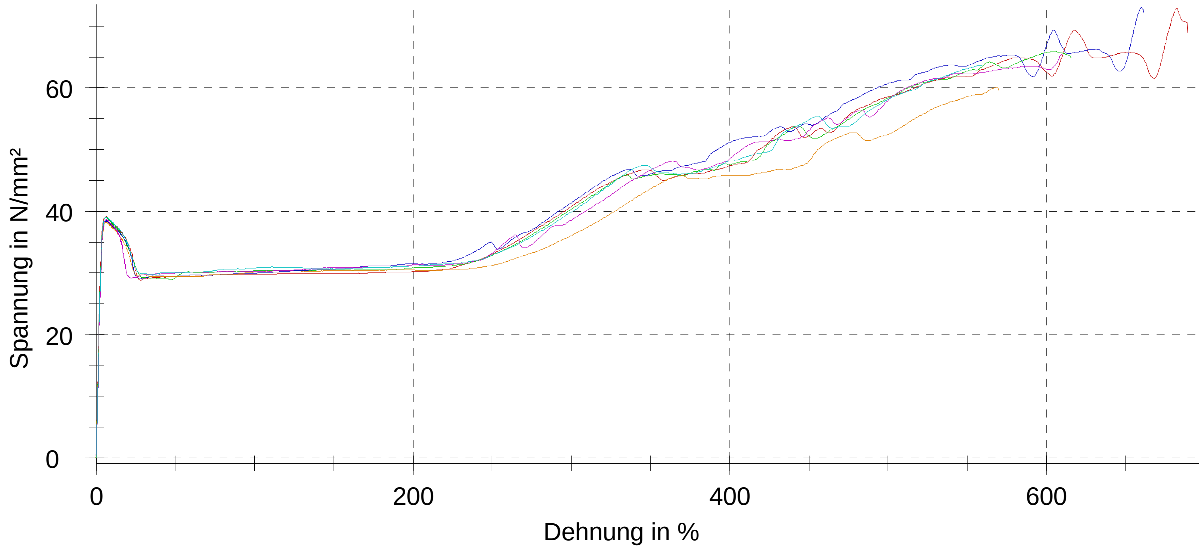

# Zugprüfung ISO 527
# Probekörper 1BA (1:2)

16.05.19

| | | |
|---|---|---|
| FOK-Nummer | : | 172314/01A |
| Rezeptur-Nr. | : | 10% OMF753 untreated |
| Kunde | : | |
| Prüfer | : | IPF |
| Material | : | EOS PA2200 |
| Füllstoff | : | |
| Maschinendaten | : | Steuerung WN: 158988<br>Traverse WN: 158988<br>Kraft 20 kN WN: 158989<br>Makro-Wegaufnehmer WN: 162140 |

| | | | |
|---|---|---|---|
| Vorkraft | : | 2 | N |
| Geschwindigkeit Zugmodul | : | 1 | mm/min |
| Prüfgeschwindigkeit | : | 100 | mm/min |
| Beginn Zugmodulermittlung | : | 0.05 | % |
| Ende Zugmodulermittlung | : | 0.25 | % |
| Messlänge Standardweg | : | 25 | mm |

## Prüfergebnisse:

| Nr | Breite B mm | Dicke A mm | E-Modul N/mm² | Streckspannung N/mm² | Streckdehnung % | Zugfestigkeit N/mm² | Dehnung Zugf. % | Bruchspannung N/mm² | Bruchdehnung % | W bis Bruch J |
|---|---|---|---|---|---|---|---|---|---|---|
| 1 | 5 | 0.98 | 1400 | 43.6 | 5.9 | 51.0 | 457.0 | 49.4 | 457.3 | 20.39 |
| ✝ 2 | 5 | 0.98 | 1360 | 44.0 | 5.9 | 44.0 | 5.9 | 32.0 | 271.5 | 10.67 |
| 3 | 5 | 0.98 | 1360 | 43.7 | 6.2 | 49.2 | 434.1 | 48.5 | 442.9 | 19.29 |
| 4 | 5 | 0.98 | 1060 | 43.6 | 6.2 | 52.3 | 477.6 | 52.3 | 478.7 | 21.57 |
| ✝ 5 | 5 | 0.98 | 1340 | 43.4 | 6.1 | 58.7 | 570.9 | 58.0 | 571.3 | 27.68 |
| 6 | 5 | 0.98 | 1430 | 42.9 | 6.4 | 47.8 | 426.0 | 47.7 | 437.3 | 19.01 |
| 7 | 5 | 0.98 | 1290 | 42.8 | 5.9 | 48.7 | 374.8 | 48.4 | 376.7 | 16.08 |
| ✝ 8 | 5 | 0.98 | 1160 | 43.2 | 6.1 | 43.2 | 6.1 | 34.7 | 320.7 | 12.92 |
| 9 | 5 | 0.98 | 1630 | 43.4 | 6.0 | 51.7 | 451.6 | 51.7 | 451.6 | 20.20 |

## Statistik:

| Serie n = 6 | Breite B mm | Dicke A mm | E-Modul N/mm² | Streckspannung N/mm² | Streckdehnung % | Zugfestigkeit N/mm² | Dehnung Zugf. % | Bruchspannung N/mm² | Bruchdehnung % | W bis Bruch J |
|---|---|---|---|---|---|---|---|---|---|---|
| $\bar{x}$ | 5 | 0.98 | 1360 | 43.3 | 6.1 | 50.1 | 436.9 | 49.7 | 440.8 | 19.43 |
| s | 0.000 | 0.000 | 188 | 0.403 | 0.2 | 1.81 | 35.4 | 1.89 | 34.5 | 1.87 |
| ν | 0.00 | 0.00 | 13.80 | 0.93 | 3.33 | 3.61 | 8.10 | 3.80 | 7.83 | 9.62 |

## Seriengrafik:

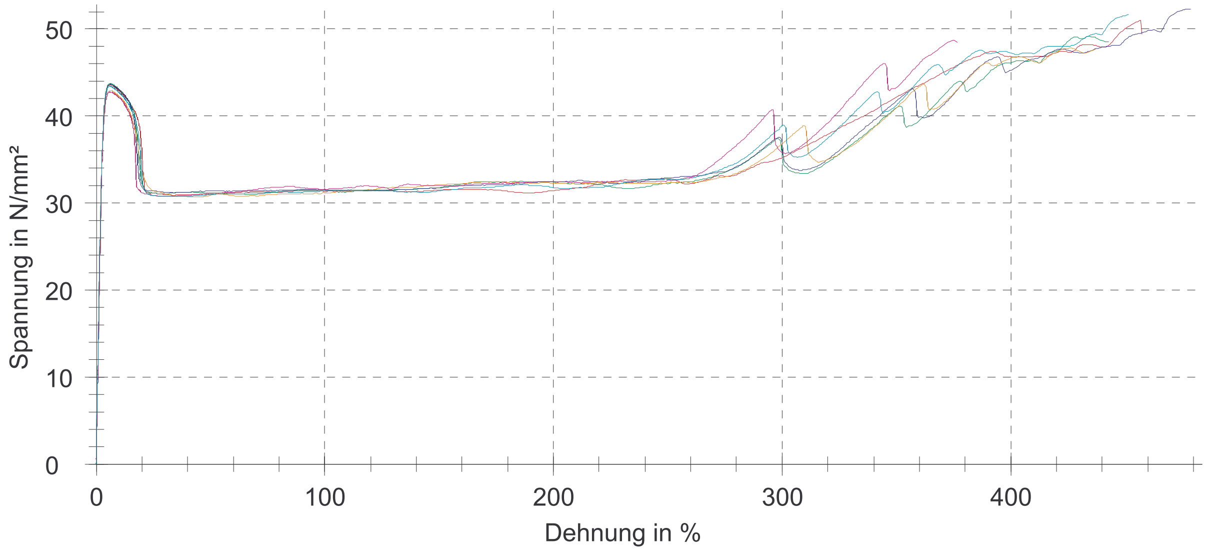

# Zugprüfung ISO 527
# Probekörper 1BA (1:2)

16.05.19

| | | |
|---|---|---|
| FOK-Nummer | : | 172314/21A |
| Rezeptur-Nr. | : | 10% OMF753 treated with 1% Aminohexanoic acid |
| Kunde | : | |
| Prüfer | : | IPF |
| Material | : | EOS PA2200 |
| Füllstoff | : | |
| Maschinendaten | : | Steuerung WN: 158988<br>Traverse WN: 158988<br>Kraft 20 kN WN: 158989<br>Makro-Wegaufnehmer WN: 162140 |

| | | | | | | | |
|---|---|---|---|---|---|---|---|
| Vorkraft | : | 2 | N | Beginn Zugmodulermittlung | : | 0.05 | % |
| Geschwindigkeit Zugmodul | : | 1 | mm/min | Ende Zugmodulermittlung | : | 0.25 | % |
| Prüfgeschwindigkeit | : | 100 | mm/min | Messlänge Standardweg | : | 25 | mm |

## Prüfergebnisse:

| Nr | Breite B mm | Dicke A mm | E-Modul N/mm² | Streckspannung N/mm² | Streckdehnung % | Zugfestigkeit N/mm² | Dehnung Zugf. % | Bruchspannung N/mm² | Bruchdehnung % | W bis Bruch J |
|---|---|---|---|---|---|---|---|---|---|---|
| 1 | 5 | 0.98 | 1220 | 41.2 | 6.1 | 57.2 | 585.2 | 55.1 | 597.2 | 28.32 |
| 2 | 5 | 0.98 | 1190 | 41.7 | 6.2 | 54.7 | 549.6 | 54.7 | 549.6 | 25.22 |
| 3 | 5 | 0.98 | 1170 | 42.0 | 5.9 | 57.6 | 524.8 | 57.6 | 525.9 | 24.87 |
| 4 | 5 | 0.98 | 1200 | 42.2 | 5.9 | 58.0 | 536.4 | 57.2 | 548.0 | 26.17 |
| 5 | 5 | 0.98 | 1260 | 41.7 | 5.9 | 50.3 | 500.6 | 50.3 | 501.2 | 21.82 |
| 6 | 5 | 0.98 | 1120 | 41.3 | 6.0 | 51.7 | 511.6 | 50.8 | 512.2 | 22.54 |

## Statistik:

| Serie n = 6 | Breite B mm | Dicke A mm | E-Modul N/mm² | Streckspannung N/mm² | Streckdehnung % | Zugfestigkeit N/mm² | Dehnung Zugf. % | Bruchspannung N/mm² | Bruchdehnung % | W bis Bruch J |
|---|---|---|---|---|---|---|---|---|---|---|
| $\bar{x}$ | 5 | 0.98 | 1190 | 41.7 | 6.0 | 54.9 | 534.7 | 54.3 | 539.0 | 24.82 |
| s | 0.000 | 0.000 | 46.6 | 0.398 | 0.1 | 3.27 | 30.2 | 3.11 | 34.4 | 2.39 |
| $\nu$ | 0.00 | 0.00 | 3.90 | 0.95 | 2.07 | 5.96 | 5.66 | 5.73 | 6.38 | 9.61 |

## Seriengrafik:

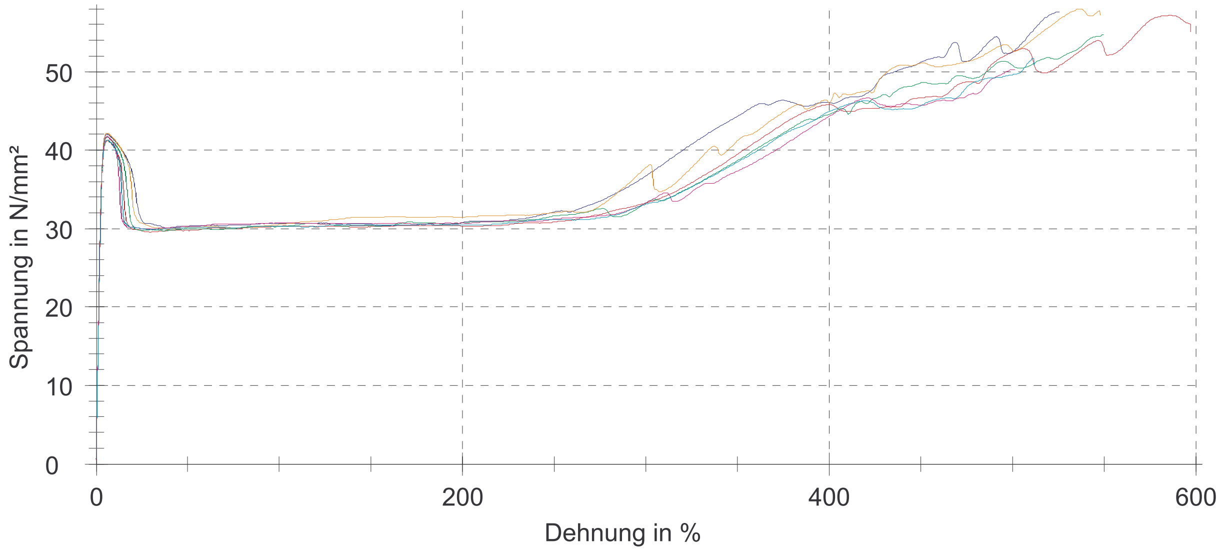

# Zugprüfung ISO 527
# Probekörper 1BA (1:2)

16.05.19

FOK-Nummer : 172314/22A
Rezeptur-Nr. : 10% OMF753 treated with 1% Aminohexanoic acid hydrolized
Kunde :
Prüfer : IPF
Material : EOS PA2200
Füllstoff :
Maschinendaten : Steuerung WN: 158988
Traverse WN: 158988
Kraft 20 kN WN: 158989
Makro-Wegaufnehmer WN: 162140

| | | | | | | |
|---|---|---|---|---|---|---|
| Vorkraft | : 2 | N | Beginn Zugmodulermittlung | : | 0.05 | % |
| Geschwindigkeit Zugmodul | : 1 | mm/min | Ende Zugmodulermittlung | : | 0.25 | % |
| Prüfgeschwindigkeit | : 100 | mm/min | Messlänge Standardweg | : | 25 | mm |

## Prüfergebnisse:

| Nr | Breite B mm | Dicke A mm | E-Modul N/mm² | Streckspannung N/mm² | Streckdehnung % | Zugfestigkeit N/mm² | Dehnung Zugf. % | Bruchspannung N/mm² | Bruchdehnung % | W bis Bruch J |
|---|---|---|---|---|---|---|---|---|---|---|
| 1 | 5 | 0.98 | 1330 | 42.0 | 5.8 | 53.2 | 526.6 | 52.4 | 533.7 | 24.45 |
| 2 | 5 | 0.98 | 1440 | 42.1 | 5.8 | 57.5 | 522.4 | 55.0 | 522.5 | 24.71 |
| 3 | 5 | 0.98 | 1210 | 41.8 | 5.8 | 58.5 | 557.3 | 58.5 | 557.3 | 26.42 |
| 4 | 5 | 0.98 | 1250 | 42.1 | 5.8 | 52.6 | 504.8 | 52.6 | 505.9 | 22.78 |
| 5 | 5 | 0.98 | 1410 | 42.8 | 5.7 | 57.6 | 527.0 | 55.5 | 527.2 | 24.97 |
| 6 | 5 | 0.98 | 1250 | 42.7 | 5.7 | 56.6 | 517.8 | 56.2 | 518.5 | 24.22 |

## Statistik:

| Serie n = 6 | Breite B mm | Dicke A mm | E-Modul N/mm² | Streckspannung N/mm² | Streckdehnung % | Zugfestigkeit N/mm² | Dehnung Zugf. % | Bruchspannung N/mm² | Bruchdehnung % | W bis Bruch J |
|---|---|---|---|---|---|---|---|---|---|---|
| $\bar{x}$ | 5 | 0.98 | 1320 | 42.3 | 5.8 | 56.0 | 526.0 | 55.0 | 527.5 | 24.59 |
| s | 0.000 | 0.000 | 92.1 | 0.407 | 0.1 | 2.46 | 17.4 | 2.31 | 17.3 | 1.18 |
| ν | 0.00 | 0.00 | 6.99 | 0.96 | 0.91 | 4.39 | 3.30 | 4.20 | 3.28 | 4.79 |

## Seriengrafik:

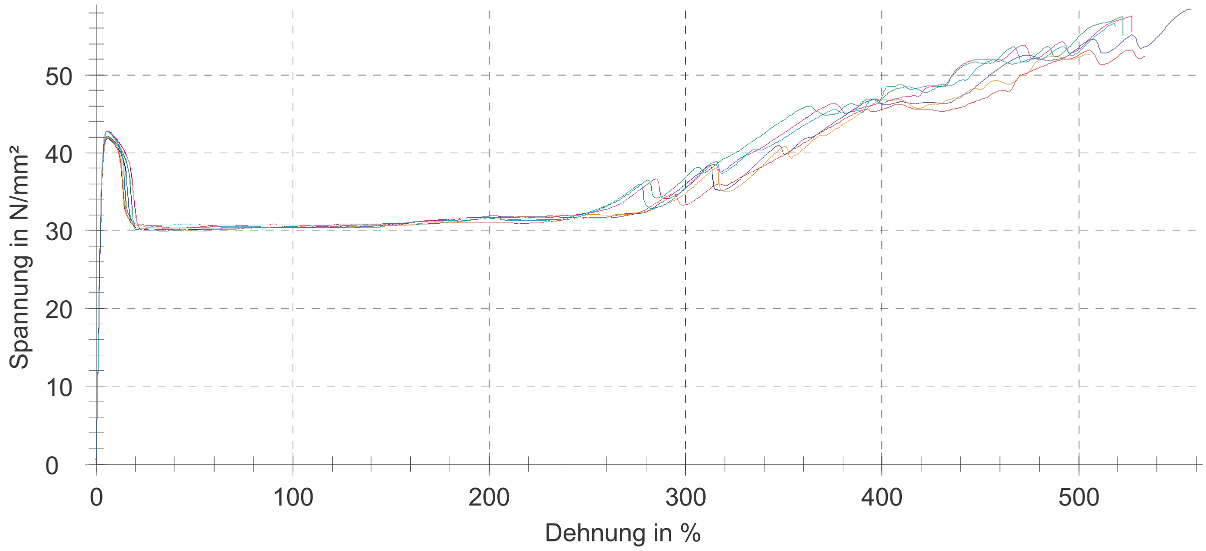

# Zugprüfung ISO 527
# Probekörper 1BA (1:2)

16.05.19

FOK-Nummer : 172314/23A
Rezeptur-Nr. : 10% OMF753 treated with 1% Caprolactam hydrolized
Kunde :
Prüfer : IPF
Material : EOS PA2200
Füllstoff :
Maschinendaten : Steuerung WN: 158988
Traverse WN: 158988
Kraft 20 kN WN: 158989
Makro-Wegaufnehmer WN: 162140

| | | | | | | |
|---|---|---|---|---|---|---|
| Vorkraft | : | 2 | N | Beginn Zugmodulermittlung | : | 0.05 % |
| Geschwindigkeit Zugmodul | : | 1 | mm/min | Ende Zugmodulermittlung | : | 0.25 % |
| Prüfgeschwindigkeit | : | 100 | mm/min | Messlänge Standardweg | : | 25 mm |

## Prüfergebnisse:

| Nr | Breite B mm | Dicke A mm | E-Modul N/mm² | Streckspannung N/mm² | Streckdehnung % | Zugfestigkeit N/mm² | Dehnung Zugf. % | Bruchspannung N/mm² | Bruchdehnung % | W bis Bruch J |
|---|---|---|---|---|---|---|---|---|---|---|
| 1 | 5 | 0.98 | 1430 | 43.1 | 5.6 | 57.8 | 520.6 | 57.8 | 520.6 | 24.69 |
| 2 | 5 | 0.98 | 1130 | 42.9 | 5.9 | 56.8 | 502.8 | 56.8 | 502.8 | 23.51 |
| 3 | 5 | 0.98 | 1210 | 42.5 | 6.0 | 56.9 | 525.1 | 56.9 | 525.1 | 24.64 |
| 4 | 5 | 0.98 | 1320 | 42.3 | 6.0 | 57.6 | 529.3 | 57.6 | 529.8 | 25.01 |
| 5 | 5 | 0.98 | 1290 | 42.2 | 6.0 | 57.6 | 543.9 | 57.6 | 543.9 | 25.61 |

## Statistik:

| Serie n = 5 | Breite B mm | Dicke A mm | E-Modul N/mm² | Streckspannung N/mm² | Streckdehnung % | Zugfestigkeit N/mm² | Dehnung Zugf. % | Bruchspannung N/mm² | Bruchdehnung % | W bis Bruch J |
|---|---|---|---|---|---|---|---|---|---|---|
| $\bar{x}$ | 5 | 0.98 | 1280 | 42.6 | 5.9 | 57.3 | 524.3 | 57.3 | 524.4 | 24.69 |
| s | 0.000 | 0.000 | 114 | 0.417 | 0.2 | 0.448 | 14.9 | 0.444 | 14.9 | 0.76 |
| ν | 0.00 | 0.00 | 8.95 | 0.98 | 2.79 | 0.78 | 2.83 | 0.77 | 2.84 | 3.09 |

## Seriengrafik:

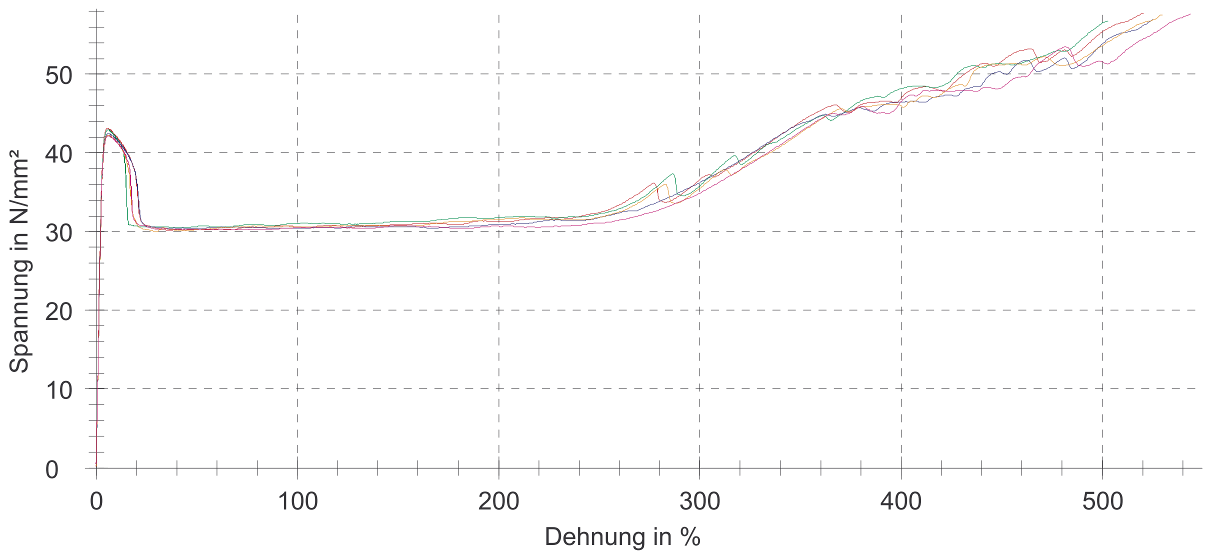

# Zugprüfung ISO 527
# Probekörper 1BA (1:2)

16.05.19

| | | |
|---|---|---|
| FOK-Nummer | : | 172314/24A |
| Rezeptur-Nr. | : | 10% OMF753 treated with 1% L-Arginine hydrolized |
| Kunde | : | |
| Prüfer | : | IPF |
| Material | : | EOS PA2200 |
| Füllstoff | : | |
| Maschinendaten | : | Steuerung WN: 158988<br>Traverse WN: 158988<br>Kraft 20 kN WN: 158989<br>Makro-Wegaufnehmer WN: 162140 |

| | | | | | | | |
|---|---|---|---|---|---|---|---|
| Vorkraft | : | 2 | N | Beginn Zugmodulermittlung | : | 0.05 | % |
| Geschwindigkeit Zugmodul | : | 1 | mm/min | Ende Zugmodulermittlung | : | 0.25 | % |
| Prüfgeschwindigkeit | : | 100 | mm/min | Messlänge Standardweg | : | 25 | mm |

## Prüfergebnisse:

| Nr | Breite B mm | Dicke A mm | E-Modul N/mm² | Streckspannung N/mm² | Streckdehnung % | Zugfestigkeit N/mm² | Dehnung Zugf. % | Bruchspannung N/mm² | Bruchdehnung % | W bis Bruch J |
|---|---|---|---|---|---|---|---|---|---|---|
| ✝ 1 | 5 | 0.98 | 961 | 42.1 | 5.4 | 50.3 | 380.3 | 49.7 | 380.9 | 16.37 |
| 2 | 5 | 0.98 | 1200 | 42.1 | 5.6 | 57.3 | 557.4 | 57.2 | 557.9 | 26.25 |
| 3 | 5 | 0.98 | 1270 | 42.1 | 5.8 | 52.2 | 490.4 | 50.5 | 491.3 | 21.92 |
| 4 | 5 | 0.98 | 1310 | 42.4 | 5.7 | 57.9 | 543.7 | 57.9 | 544.2 | 25.85 |
| 5 | 5 | 0.98 | 1540 | 43.0 | 5.6 | 56.0 | 541.6 | 55.4 | 541.6 | 25.50 |
| 6 | 5 | 0.98 | 1310 | 43.0 | 5.6 | 58.0 | 520.6 | 58.0 | 520.6 | 24.97 |
| 7 | 5 | 0.98 | 1500 | 42.9 | 5.7 | 58.2 | 523.6 | 56.0 | 523.7 | 24.98 |
| ✝ 8 | 5 | 0.98 | 1410 | 43.2 | 5.7 | 50.1 | 455.9 | 50.0 | 457.0 | 20.16 |
| 9 | 5 | 0.98 | 1490 | 43.0 | 5.5 | 56.8 | 543.1 | 56.0 | 544.6 | 26.11 |

## Statistik:

| Serie n = 7 | Breite B mm | Dicke A mm | E-Modul N/mm² | Streckspannung N/mm² | Streckdehnung % | Zugfestigkeit N/mm² | Dehnung Zugf. % | Bruchspannung N/mm² | Bruchdehnung % | W bis Bruch J |
|---|---|---|---|---|---|---|---|---|---|---|
| $\bar{x}$ | 5 | 0.98 | 1370 | 42.7 | 5.6 | 56.6 | 531.5 | 55.9 | 532.0 | 25.08 |
| s | 0.000 | 0.000 | 132 | 0.451 | 0.1 | 2.11 | 22.1 | 2.58 | 22.1 | 1.48 |
| ν | 0.00 | 0.00 | 9.64 | 1.06 | 1.46 | 3.73 | 4.16 | 4.62 | 4.15 | 5.91 |

## Seriengrafik:

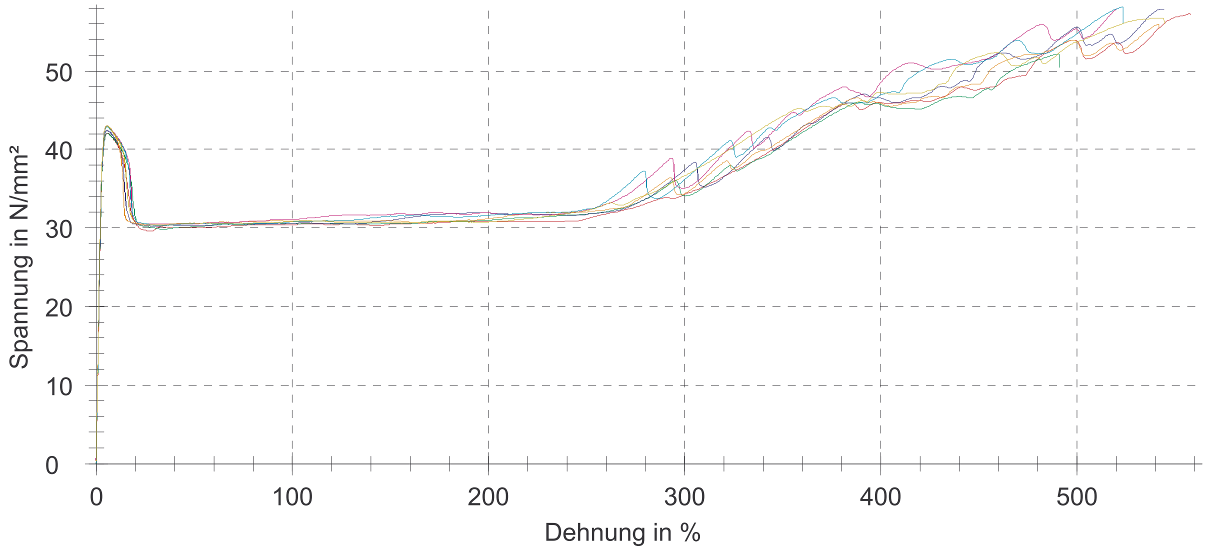

# Zugprüfung ISO 527
# Probekörper 1BA (1:2)

16.05.19

| | | |
|---|---|---|
| FOK-Nummer | : | 172314/25A |
| Rezeptur-Nr. | : | 10% OMF753 treated with 1% Glutamic acid Na-salt |
| Kunde | : | |
| Prüfer | : | IPF |
| Material | : | EOS PA2200 |
| Füllstoff | : | |
| Maschinendaten | : | Steuerung WN: 158988<br>Traverse WN: 158988<br>Kraft 20 kN WN: 158989<br>Makro-Wegaufnehmer WN: 162140 |

| | | | | | | | |
|---|---|---|---|---|---|---|---|
| Vorkraft | : | 2 | N | Beginn Zugmodulermittlung | : | 0.05 | % |
| Geschwindigkeit Zugmodul | : | 1 | mm/min | Ende Zugmodulermittlung | : | 0.25 | % |
| Prüfgeschwindigkeit | : | 100 | mm/min | Messlänge Standardweg | : | 25 | mm |

## Prüfergebnisse:

| Nr | Breite B mm | Dicke A mm | E-Modul N/mm² | Streckspannung N/mm² | Streckdehnung % | Zugfestigkeit N/mm² | Dehnung Zugf. % | Bruchspannung N/mm² | Bruchdehnung % | W bis Bruch J |
|---|---|---|---|---|---|---|---|---|---|---|
| 1 | 5 | 0.98 | 1240 | 44.0 | 5.8 | 49.7 | 429.4 | 49.7 | 429.9 | 18.97 |
| 2 | 5 | 0.98 | 1470 | 44.0 | 5.9 | 54.9 | 493.5 | 52.2 | 494.4 | 23.03 |
| ✝ 3 | 5 | 0.98 | 1170 | 43.9 | 5.9 | 57.7 | 528.8 | 57.2 | 529.0 | 25.56 |
| ✝ 4 | 5 | 0.98 | 1410 | 44.0 | 5.9 | 57.2 | 505.4 | 57.2 | 505.4 | 23.75 |
| 5 | 5 | 0.98 | 1310 | 43.9 | 5.9 | 56.1 | 482.0 | 54.5 | 482.6 | 22.71 |
| 6 | 5 | 0.98 | 1360 | 44.1 | 5.7 | 54.9 | 477.0 | 54.9 | 477.5 | 22.35 |
| 7 | 5 | 0.98 | 1520 | 44.5 | 5.7 | 54.7 | 445.3 | 54.3 | 445.9 | 20.74 |
| ✝ 8 | 5 | 0.98 | 1290 | 44.6 | 5.8 | 58.8 | 495.6 | 53.4 | 501.1 | 24.26 |
| 9 | 5 | 0.98 | 1320 | 44.6 | 5.8 | 52.8 | 438.4 | 52.3 | 445.7 | 20.47 |

## Statistik:

| Serie n = 6 | Breite B mm | Dicke A mm | E-Modul N/mm² | Streckspannung N/mm² | Streckdehnung % | Zugfestigkeit N/mm² | Dehnung Zugf. % | Bruchspannung N/mm² | Bruchdehnung % | W bis Bruch J |
|---|---|---|---|---|---|---|---|---|---|---|
| $\bar{x}$ | 5 | 0.98 | 1370 | 44.2 | 5.8 | 53.9 | 460.9 | 53.0 | 462.6 | 21.38 |
| s | 0.000 | 0.000 | 105 | 0.306 | 0.1 | 2.29 | 26.5 | 1.96 | 25.6 | 1.58 |
| ν | 0.00 | 0.00 | 7.66 | 0.69 | 1.52 | 4.26 | 5.75 | 3.71 | 5.53 | 7.39 |

## Seriengrafik:

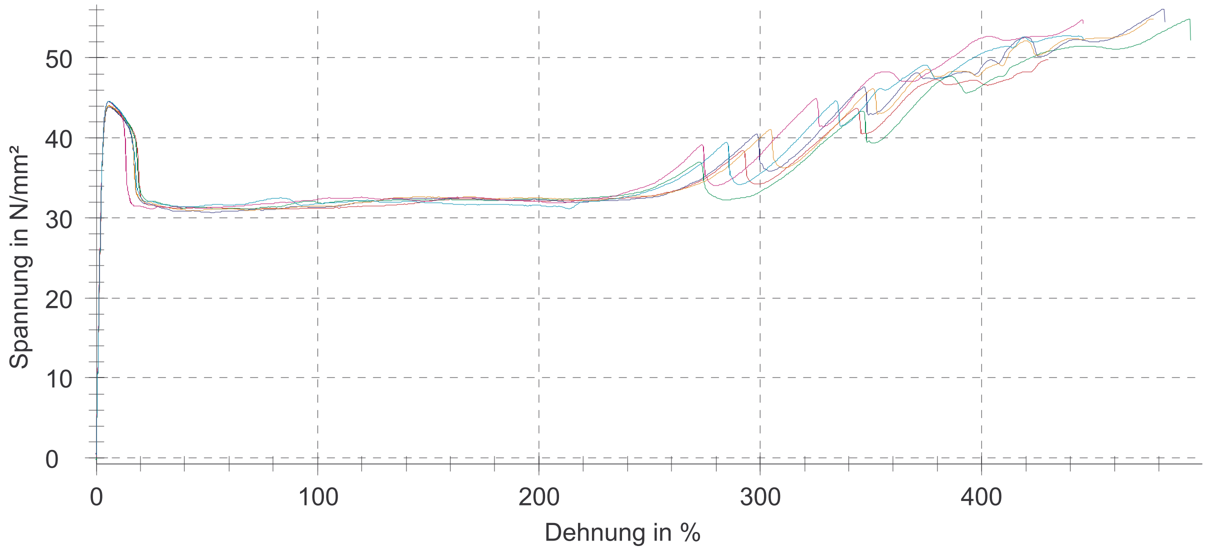

# Zugprüfung ISO 527
# Probekörper 1BA (1:2)

16.05.19

FOK-Nummer : 172314/26A
Rezeptur-Nr. : 10% OMF753 treated with 1% ASA
Kunde :
Prüfer : IPF
Material : EOS PA2200
Füllstoff :
Maschinendaten : Steuerung WN: 158988
Traverse WN: 158988
Kraft 20 kN WN: 158989
Makro-Wegaufnehmer WN: 162140

Vorkraft : 2 N
Geschwindigkeit Zugmodul : 1 mm/min
Prüfgeschwindigkeit : 100 mm/min
Beginn Zugmodulermittlung : 0.05 %
Ende Zugmodulermittlung : 0.25 %
Messlänge Standardweg : 25 mm

## Prüfergebnisse:

| Nr | Breite B mm | Dicke A mm | E-Modul N/mm² | Streckspannung N/mm² | Streckdehnung % | Zugfestigkeit N/mm² | Dehnung Zugf. % | Bruchspannung N/mm² | Bruchdehnung % | W bis Bruch J |
|---|---|---|---|---|---|---|---|---|---|---|
| ✝ 1 | 5 | 0.98 | 1540 | 43.1 | 5.9 | 57.0 | 523.1 | 56.8 | 524.2 | 24.80 |
| 2 | 5 | 0.98 | 1400 | 43.0 | 5.8 | 51.9 | 444.4 | 50.6 | 468.0 | 21.09 |
| ✝ 3 | 5 | 0.98 | 1200 | 43.1 | 5.9 | 56.1 | 540.3 | 55.3 | 540.4 | 25.68 |
| ✝ 4 | 5 | 0.98 | 1330 | 42.8 | 6.0 | 55.3 | 543.4 | 53.2 | 543.7 | 25.27 |
| ✝ 5 | 5 | 0.98 | 1270 | 42.9 | 5.9 | 57.4 | 554.3 | 54.8 | 554.5 | 26.33 |
| 6 | 5 | 0.98 | 1150 | 43.4 | 6.1 | 54.7 | 488.1 | 54.7 | 488.1 | 22.15 |
| 7 | 5 | 0.98 | 1640 | 43.1 | 6.1 | 52.0 | 479.3 | 52.0 | 479.3 | 21.43 |
| 8 | 5 | 0.98 | 1160 | 42.9 | 6.0 | 52.2 | 478.4 | 52.0 | 479.4 | 21.22 |
| 9 | 5 | 0.98 | 1460 | 43.0 | 6.3 | 55.0 | 487.2 | 53.3 | 487.5 | 22.41 |

## Statistik:

| Serie n = 5 | Breite B mm | Dicke A mm | E-Modul N/mm² | Streckspannung N/mm² | Streckdehnung % | Zugfestigkeit N/mm² | Dehnung Zugf. % | Bruchspannung N/mm² | Bruchdehnung % | W bis Bruch J |
|---|---|---|---|---|---|---|---|---|---|---|
| $\bar{x}$ | 5 | 0.98 | 1360 | 43.1 | 6.1 | 53.2 | 475.5 | 52.5 | 480.5 | 21.66 |
| s | 0.000 | 0.000 | 208 | 0.194 | 0.2 | 1.57 | 17.9 | 1.58 | 8.1 | 0.59 |
| ν | 0.00 | 0.00 | 15.29 | 0.45 | 3.19 | 2.96 | 3.77 | 3.01 | 1.69 | 2.72 |

## Seriengrafik:

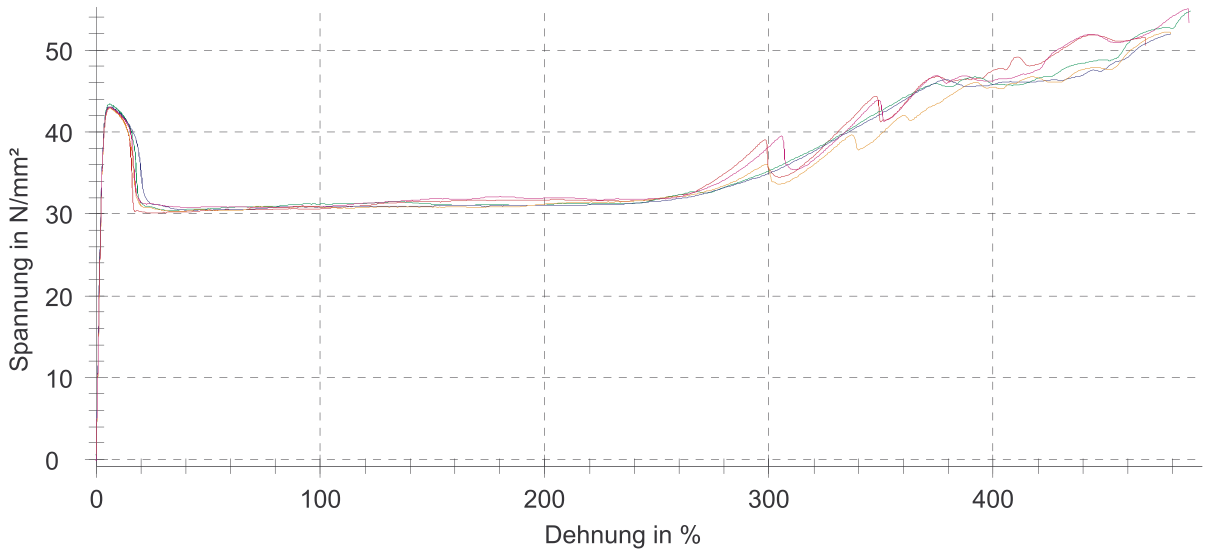

Supplement: Supplementary file 1 [file polymers-12-01295-s001.pdf]
